# EHMTI-0299. Serotonin as biomarker of ache intensity in chronic tension headache

**DOI:** 10.1186/1129-2377-15-S1-C27

**Published:** 2014-09-18

**Authors:** I Karakulova

**Affiliations:** 1 Department of Neurology, Perm State Medical Academy, Perm, Russia

## Introduction

Tension headache (TH) belongs to most frequent type of the idiopathic cephalalgies. Depression and serotonins metabolism disorders play a leading role in the pathogenesis of TH.

## Aims

Study the dependence of the intensity of the TH of degree of depression, anxiety and levels of serum serotonin.

## Methods

The complex algic, psychometric test and determination of concentration of blood serum serotonin (S) by the method of immunoenzyme analysis were performed in 140 patients with TH.

## Results

At patients of first group the intensity of episodic TH (44 man) was 56±4,32 mm according to 100-mm visual analogue scale. All patients of this group had high level of reactive anxiety (50,18±8,6, p<0,05), moderate level of depression and tendency to the decreasing of blood S concentration (205,72±21,62 ng/ml). The second group consists of 96 patients with chronic TH. Straight correlation between duration and intensity of ache, indices of reactive and personal anxiety (46,81±8,8 and 54,2+9,64 accordingly), high depression level (23,6±4,52 points in according with Bek scale, p=0,001) and significant decreasing of blood S concentration (119,38±19,42 ng/ml, p=0,046) was determined in all patients of this group.

## Conclusions

Concentration of blood serum serotonin depends on duration and intensity of ache, level of depression and anxiety. Therefore, serotonin of blood serum can be used as biomarker of ache intensity, level of depression.

No conflict of interest.

